# Exploring Bird Gut Microbiota Through Opportunistic Fecal Sampling: Ecological and Evolutionary Perspectives

**DOI:** 10.1002/ece3.71291

**Published:** 2025-04-14

**Authors:** Laura Fablet, Aurélie Bonin, Diane Zarzoso‐Lacoste, Vincent Dubut, Laurence Walch

**Affiliations:** ^1^ Sorbonne Université, CNRS, IRD, INRAE Université Paris Est Créteil, Université Paris Cité, Institute of Ecology and Environmental Sciences (IEES‐Paris) Paris France; ^2^ ARGALY Sainte Hélène du Lac France; ^3^ UMR CNRS 7058 Ecologie et Dynamique Des Systèmes Anthropisés (EDYSAN) Université de Picardie Jules Verne Amiens France; ^4^ Aix Marseille Univ Avignon Université, CNRS, IRD, IMBE Marseille France; ^5^ ADENEKO Saint‐Girons France

**Keywords:** avian health, bacterial composition, bird communities, freshwater ecosystems, molecular ecology

## Abstract

Wetland ecosystems are facing alarming rates of destruction and degradation, posing significant challenges for avian populations reliant on these habitats. Bird health is closely linked to the composition of their intestinal microbiota, which is primarily influenced by local conditions, primarily through diet. Building on our previous work identifying dietary variations among bird populations in marshes within a Ramsar site along the Somme and Avre rivers (France), this pilot study aimed to assess the relevance of using fecal samples collected from the ground to characterize avian intestinal microbiota via 16S rRNA metabarcoding. We hypothesized that this noninvasive sampling method would capture how bird traits and environmental factors shape fecal microbiota composition. Sampling was conducted during the breeding season at seven locations (six within the Ramsar site and one on its outskirts) spanning rural or peri‐urban environments. A total of 52 fecal samples from nine bird species or families, predominantly waterbirds, were analyzed for bacterial composition. At the phylum level, Firmicutes and Proteobacteria were predominant, with the relative abundance of genera such as *Clostridium*, *Rothia*, *Bacillus*, *Caldilinea* and *Pseudomonas* varying among bird species. The potential enteropathogen *Campylobacter* was primarily detected in samples from peri‐urban sites. Multivariate analyses revealed significant variations in bacterial composition associated with bird trophic guild, ecology, body length, pond surface and habitat location. Additionally, a weak correlation was observed between host phylogeny and microbiota composition. Although the limited sample size, particularly for some species, constrains the robustness of these findings, the observed trends align with ecological expectations. This study highlights the potential of opportunistically collected fecal samples as a low‐impact tool for exploring the relationship between bird gut microbiota and their habitat.

## Graphical abstract

Wetland degradation threatens bird populations, and their intestinal microbiota composition is influenced by local conditions, particularly diet. This pilot study used fecal samples collected from the ground to assess avian gut microbiota via 16S rRNA metabarcoding, revealing significant variations linked to bird traits and environmental factors. Despite limitations in sample size, the findings support the use of noninvasive fecal sampling as a valuable tool for studying bird microbiota in relation to habitat.

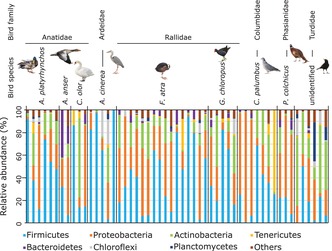

## 1 Introduction

The gut microbiota of birds plays a crucial role in various physiological processes, including gut development, digestion, pathogen exclusion, immune function and behavior (Waite and Taylor 2014; Grond et al. 2018; Sun et al. 2022). Additionally, the plasticity of the gut metagenome is thought to facilitate rapid ecological acclimation and adaptation of the host organism to environmental changes (Alberdi et al. 2016). Microbial communities associated with hosts follow the same ecological principles as macro‐ecological systems (Costello et al. 2012), where microbial interactions (e.g., competition, cooperation, etc.) and environmental factors shape gut bacterial composition. These factors may be intrinsic to the host, such as genetics, age, sex, trophic guild, and health status, or extrinsic, including diet, social interactions and exposure to environmental microbial sources (Grond et al. 2018).

Among intrinsic factors, host phylogeny is generally considered a weak predictor of gut microbiota diversity in birds (Capunitan et al. 2020; Song et al. 2020), in contrast to mammals, where cophylogeny strongly influences microbiota composition (Youngblut et al. 2019). This discrepancy may be partly explained by adaptations to flight, as suggested by the convergence of gut microbiota in birds and bats (Song et al. 2020). The high metabolic demands of flight likely impose selective pressures favoring microbiota that optimize energy extraction while minimizing digestive burden. Birds and bats also exhibit short gut retention times due to morphological adaptations, such as a relatively short intestinal tracts, which may further influence microbiota composition (Song et al. 2020). In passerines, Bodawatta, Koane, et al. (2021) reported a negative correlation between gut retention time and both microbial diversity and cloacal microbiota heterogeneity across 17 species.

By contrast, trophic guild has been identified as a strong predictor of gut microbiota composition in birds (Bodawatta, Koane, et al. 2021; Jarma et al. 2021; Wang, Zhong, et al. 2022). In vertebrates, dietary preferences shape microbial functional guilds that aid in host nutrition (Youngblut et al. 2019). In birds, a comparative study found that herbivorous and carnivorous migratory waterbirds harbor less diverse and complex microbial networks than omnivores, with lower integration and stability (Wang, Zhong, et al. 2022). However, the association between specific bacterial taxa and trophic guilds remains complex and not exclusive. While general patterns exist (e.g., fiber‐digesting bacteria in herbivores), functional redundancy and interindividual variability complicate the identification of strictly guild‐specific taxa. Furthermore, a bird's ecological niche may contribute to this complexity, as habitat use (e.g., aquatic, terrestrial and arboreal) has been shown to influence gut microbiota composition across avian orders (Wang, Hong, et al. 2022; Jones et al. 2023). However, in some of these studies, interpretation is challenging because sampling was conducted in captivity (e.g., zoos), where microbiota composition may differ significantly from that of wild populations (Wienemann et al. 2011; Xie et al. 2016).

Among the extrinsic factors related to the ecological niche, diet is a major driver of gut microbiota structure. Experimental studies in passerines have demonstrated that diet influences microbial diversity, taxonomic composition and functional profile (Davidson et al. 2020; Teyssier et al. 2020; Bodawatta, Freiberga, et al. 2021). The transfer of bacteria through trophic networks, from primary producers to secondary consumers, contributes to microbiota variation in birds (Dion‐Phénix et al. 2021; Bodawatta, Klečková, et al. 2022). However, the strength of this effect varies across species, underscoring the complexity of these interactions (Perry et al. 2017; Davidson et al. 2020; Teyssier et al. 2020; Bodawatta, Freiberga, et al. 2021). Gut microbiota composition can also shift seasonally in response to dietary and habitat changes associated with migration, reproduction or environmental fluctuations (Tang et al. 2023; Zhang et al. 2025).

Anthropogenic activities further influence avian diet and, consequently, gut microbiota. Habitat conversion, such as deforestation for agriculture, often degrades habitat quality, leading to shifts in passerine microbiota composition and diversity, although species‐specific vulnerabilities exist (Teyssier et al. 2018, 2020; San Juan et al. 2020). Similarly, wetland loss and land‐use changes have forced many waterbirds to rely on artificial habitats (e.g., urban wetlands, wastewater treatment areas, polluted environments, landfill sites), where altered food availability, including human‐provided resources, impacts their diet (Murray and Hamilton 2010; Brochet et al. 2012; Wang, Kuang, et al. 2018; Evans and Gawlik 2020; Murray et al. 2020). Several studies suggest that exposure to these environments alters waterbird gut microbiota and increases colonization by potential bacterial pathogens (Murray et al. 2020; Jarma et al. 2021; Wang et al. 2023). For example, the critically endangered Siberian Crane (*Leucogeranus leucogeranus*) has recently shifted its diet in southern China, replacing aquatic tubers with agricultural crops. This dietary transition has resulted in a microbiota shift, with tuber‐feeding cranes exhibiting an enrichment in fiber‐digesting Clostridiaceae, while crop‐feeding individuals harbor a more diverse microbiota, including carbohydrate‐metabolizing bacteria and potential pathogens (Wang et al. 2023). Similarly, urbanized American white ibises ( *Eudocimus albus* ) that consume carbohydrate‐rich provisioned bread instead of high‐protein natural food exhibit significant gut microbiota shifts, potentially increasing their susceptibility to enteric pathogens such as *Salmonella* (Murray et al. 2020).

As ecosystems undergo rapid environmental changes, conservation biology has become increasingly relevant in the Anthropocene. Recent research highlights the importance of gut microbiota studies for conservation efforts (Trevelline et al. 2019; Sehnal et al. 2021). Gut microbiota analysis can identify dysbioses, which are microbial imbalances associated with various factors, including environmental disturbances, and can help develop new metrics for assessing habitat quality and restoration effectiveness. Understanding how habitat quality influences microbial community composition and pathogen transmission in wild birds could provide valuable insights for conservation strategies (Bodawatta, Hird, et al. 2022).

This study aimed to assess the feasibility of investigating factors shaping avian gut microbiota composition in a wetland ecosystem using 16S rRNA metabarcoding and a noninvasive, opportunistic fecal sampling approach, wherein bird droppings were collected directly from the ground without species identification or precise knowledge of deposition time. Specifically, we evaluated whether this method could capture the influence of phylogeny, host traits (body length as a proxy of gut length, ecology: terrestrial vs. aquatic, and trophic guild), and environmental factors (diet, habitat type: urban vs. rural, and habitat structure: pond size) on microbiota structure. This approach could provide a promising, non‐invasive tool for large‐scale ecological and evolutionary studies, particularly in critical conservation areas where traditional sampling methods may be impractical.

## 2 Materials and Methods

### 2.1 Field Description

**FIGURE 1:**
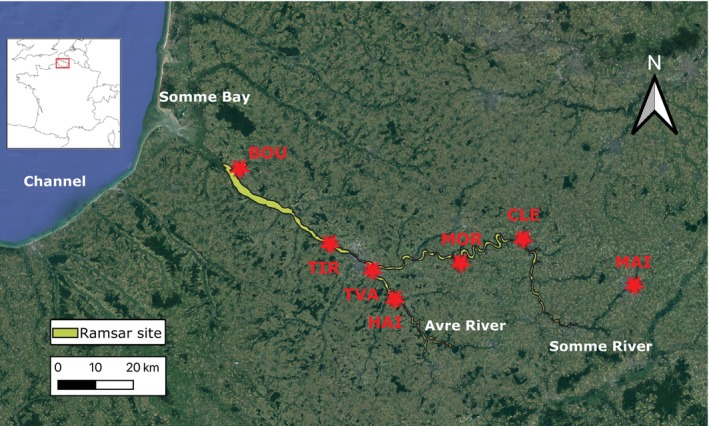
Sampling sites. The seven marshes included in this study are located in Northern France along the Channel, an arm of the Atlantic Ocean separating Southern England from Northern France. The French administrative department of the Somme (outlined in red in the inset map of France) includes two Ramsar sites: The “Bay of Somme”, a natural estuary, and the “Marshes and Peatlands of the Somme and Avre Valleys”, shown in light green on the map. The latter encompasses the lower stretches of the Somme River and its main tributary, the Avre River, as well as adjacent marshes and peatlands. Sampling sites are predominantly located within these marshes, except for the National Nature Reserve “*Marais d'Isles*”, situated within the urban area of Saint‐Quentin. BOU, Parc de la Bouvaque; CLE, Marais de Cléry‐sur‐Somme and MAI, Marais d'Isle; HAI, Marais de Hailles; MOR, Marais de Morcourt; TIR, Marais de Tirancourt; TVA, Marais des Trois Vaches.

The study area, located in Northern France (Atlantic Biogeographic Region), encompasses wetlands in the lower reaches of the Somme and the Avre Rivers (Figure 1). Covering 13,100 ha, this Ramsar‐designed site “Marshes and Peatlands of the Somme and Avre Valleys” is one of the largest alkaline peatlands in Western Europe (François 2023) and supports significant biodiversity (Stroud and Davidson 2021).

Unlike the Picardy Littoral (Bay of Somme), which hosts over 20,000 waterbirds (Ramsar Criterion 5), the inland Somme harbors smaller populations (Schmaltz et al. 2020), including species listed on the national Red List, such as the vulnerable common teal ( *Anas crecca* ) and Eurasian bittern ( *Botaurus stellaris* ), as well as the endangered little bittern ( *Ixobrychus minutus* ) and Savi's warbler ( *Locustella luscinioides* ) (see: https://www.somme.fr/plan‐gestion‐ramsar/). These wetlands act as biodiversity reservoirs amid intense human activities such as hunting, fishing, hiking and canoeing. According to Corine Land Cover cartography, the site primarily consists of water bodies and marshes (44%), with significant urban pressure and intensive agriculture nearby.

Six marshes within the Ramsar site were monitored, varying in human impact levels (Table 1) and in waterbird population richness and density, along with a nearby National Nature Reserve (“Marais d'Isle”). These included two urban parks (Marais des Trois Vaches and Parc de la Bouvaque), a large rural pond (Marais de Cléry‐sur‐Somme) and three smaller, more isolated marshes (Marais de Hailles, Marais de Morcourt, Marais de Tirancourt). According to the collaborative wildlife observation database *Clicnat*, managed by the NGO “Picardie Nature” (Amiens, France) (https://clicnat.fr), the main breeding waterbird species at the sampling sites belong to the families Anatidae (e.g., common teal, *Anas crecca* ; common pochard, *Aythya ferina* ; and mallard and *Anas platyrhynchos* ), Ardeidae (e.g., great egret, *Ardea alba* ; gray heron, *Ardea cinerea* ; and little bittern, *Ixobrychus minutus* ) and Rallidae (e.g., common moorhen, *Gallinula chloropus* ; and Eurasian coot *Fulica atra* ). For a more comprehensive species list, see Fablet et al. (2024).

**TABLE 1:** Information on sampling sites. The seven sampled marshes vary in geographic location (rural or urban), anthropic use, and pond surface areas, providing insights into habitat diversity.

Site name	Abbreviation	Town	Geographic location	Geographical setting	River	Anthropic usage	Total pond surface (ha)
Parc de la Bouvaque	BOU	Abbevile	50°6′59″ N, 1°50′34″ E	Peri‐urban	Scardon	Urban park, fishing	7.7
Marais de Cléry‐sur‐Somme	CLE	Cléry‐sur‐Somme	49°57′14″ N, 2°53′44″ E	Rural	Somme	Waterfowl hunting, fishing	46.2
Marais de Hailles	HAI	Hailles	49°48′31″ N, 2°25′23″ E	Rural	Avre	Waterfowl hunting, fishing	7.1
Marais d'Isle	MAI	Saint‐Quentin	49°50′36″ N, 3°18′22″ E	Peri‐urban	Somme	Urban park, National Nature Reserve	14.6
Marais de Morcourt	MOR	Morcourt	49°53′47″ N, 2°39′53″ E	Rural	Somme	Waterfowl hunting, fishing	12.4
Marais de Tirancourt	TIR	Tirancourt	49°56′23″ N, 2°10′52″ E	Rural	Somme	Waterfowl hunting	9.2
Marais des Trois Vaches	TVA	Amiens	49°52′38″ N, 2°20′22″ E	peri‐urban	Avre	Urban park, fishing	12.0

### 2.2 Sample Collection

Fecal bacteria are widely used as a proxy for intestinal microbiota to avoid animal sacrifice. Fecal microbiota is considered to be a mixture of bacteria from different sections of the gastrointestinal tract and can also be influenced by environmental contamination (Grond et al. 2018). During the breeding season (April 19–30, 2021), we opportunistically collected 146 bird droppings across marshes, sampling each site only once to minimize disturbance (see Table 2). To reduce pseudo‐replication, in areas with higher dropping densities, we sampled at points separated by at least 2 m (Jarma et al. 2024). Samples were collected between 8:00 a.m. and 12:00 p.m., when temperature ranged from 3°C to 11°C. Fresh scats were selected based on appearance (dry or moist, intact) using non‐talc gloves and sterile cotton swabs, then placed in plastic bags, immediately stored in a cooler, and frozen at −20°C the same evening. They were subsequently preserved at −80°C until DNA extraction. Field conditions and storage methods can significantly impact the results of 16S rRNA gene‐based analyses of bird fecal microbiota, particularly affecting the relative abundance of dominant phyla such as Firmicutes and Proteobacteria (Vargas‐Pellicer et al. 2019).

**TABLE 2:** Number of samples included in the analysis per bird species and sampling sites. Bird species were previously identified using metabarcoding (Fablet et al. 2024).

Site	Number of samples								
	*Anas platyrhynchos*	*Anser anser*	*Ardea cinerea*	*Columba palumbus*	*Cygnus olor*	*Fulica atra*	*Gallinula chloropus*	*Phasianus colchicus*	Turdidae
BOU	2			3		9	1		
CLE	1		1	2		4	3		
HAI								1	2
MAI	1		1	2	1	3	1		2
MOR		2			1			2	2
TIR			1						
TVA	2		2						

Bird species were identified via metabarcoding analysis of fecal samples. Although we did not conduct a standardized bird survey before sampling, we recorded bird species observed during fieldwork. Except for the common pheasant, all species detected by metabarcoding were also directly observed. Additionally, as detailed in Fablet et al. (2024), each fecal specimen was photographed and compared to a field guide to bird traces, confirming consistency between visual identification and metabarcoding results. Fledglings were rarely observed, and we did not sample directly beneath nests. The size and appearance of the collected feces were consistent with those of adult birds.

### 2.3 DNA Extraction, Bird Species Identification (12S rRNA) and Bacterial Microbiota Analysis (16S rRNA) Using High‐Throughput Sequencing

We extracted DNA using the DNeasy mericon Food kit (Qiagen, Germany) (Fablet et al. 2024), with mechanical lysis via nitrogen freezing and 3 mm zirconium beads (Fisher Scientific, United States) in a Mixer Mill MM 400 (Retsch GmbH, Germany) for 2 × 45 s at 30 Hz. To minimize contamination, we extracted as much of the inner fecal material as possible, avoiding the outer layer exposed to the environment (Jarma et al. 2024). Of the 146 samples, DNA extraction was successful for 116, while the remaining samples were insufficient or lost due to a technical issue. Among the 116 samples, 74 were successfully amplified by PCR for bird species identification using the MiBird‐U primer pair (Table 3). Thermocycling conditions consisted of an initial denaturation (15 min at 95°C), followed by 35 cycles (30 s at 94°C, 40 s at 50°C, and 60 s at 72°C), and a final extension (10 min at 72°C). Low amplification success was largely attributed to the presence of uric acid in the bird feces, which inhibited the PCR reaction (Fablet et al. 2024).

**TABLE 3:** Sequences and annealing temperatures of DNA primer pairs used for PCR. Degenerate bases are marked in bold. Primer pairs were designed to amplify specific regions, including the avian mitochondrial 12S rRNA gene and the bacterial V4 hypervariable region of the 16S rRNA gene.

Primer name	Target taxa	Target gene	Primer type	Primer sequence (5′–3′)	Annealing temperature (°C)	Source
MiBird‐U‐F	Birds	mt 12S rRNA	Forward	GGGTTGGTAAATCTTGTGCCAGC	50	Ushio et al. (2018)
MiBird‐U‐R	Birds	mt 12S rRNA	Reverse	CATAGTGGGGTATCTAATCCCAGTTTG	50	Ushio et al. (2018)
515FY	Bacteria	16S rRNA V4 region	Forward	GTG**Y**CAGC**M**GCCGCGGTAA	53	Parada et al. (2016)
806RB	Bacteria	16S rRNA V4 region	Reverse	GGACTAC**NV**GGGT**W**TCTAAT	53	Apprill et al. (2015)

Microbial diversity was assessed using primers targeting the V4 hypervariable region of the bacterial 16S rRNA gene: 515FY (forward) and 806RB (reverse) (Table 3; Apprill et al. 2015; Parada et al. 2016). Each DNA extract underwent triplicate PCR amplification in a 25 μL volume using the QIAGEN Multiplex PCR kit (Qiagen, Germany). Thermocycling conditions for bacterial 16S amplification included an initial denaturation (15 min at 95°C), followed by five cycles (30 s at 94°C, 40 s at 50°C, and 60 s at 72°C), then 30 cycles (30 s at 94°C, 40 s at 53°C, and 60 s at 72°C), and a final extension (10 min at 72°C). PCR products were pooled in equal volumes and purified with the Wizard SV Gel and PCR Clean‐Up Systems (Promega Corporation, United States).

The experiment included several controls: two negative DNA extraction controls (CEXT), two aerosol controls (TAER), and two PCR controls (CPCR) to monitor contamination (See Table S1; Corse et al. 2017). Additionally, to track false positives caused by tag jumps (Schnell et al. 2015), three bioinformatics blanks (BLNK; combinations of unused tags) were included, along with a positive control (CPOS), consisting of a mock community from the NGS Standard: 20 Strain Even Mix Genomic Material—MSA‐1002 (ATCC, United States; see Table S1).

For high‐throughput sequencing, a two‐step, tailed PCR approach was used to construct paired‐end, ready‐to‐pool amplicon libraries (Illumina Inc., United States). Library preparation, qualification, quantification, and sequencing were conducted at iGenSeq ICM (Paris Brain Institute, France) using an Illumina NovaSeq 6000 System (Illumina Inc., United States) with an SP‐500 cycle cartridge (2 × 250 bp, 800 million paired‐end reads). Per‐base sequence quality plots for the bacterial dataset are shown in Figure S1.

### 2.4 Bioinformatics Analysis of Metabarcoding Data

The raw sequencing data is publicly available at https://doi.org/10.5281/zenodo.14883869. Sequencing data were analyzed at the Argaly platform (Sainte‐Hélène‐du‐lac, France). Raw paired‐end reads were assembled, assigned to the corresponding PCR replicate, and dereplicated using the OBITools v.4 (Boyer et al. 2016) program suite. At this stage, files from the three libraries were concatenated, and sequences containing undetermined nucleotides, those shorter than 18 bp (outside the length range observed *in silico*), and those observed only once in the entire dataset were filtered out. The remaining sequences were clustered in Molecular Operational Taxonomic Units (MOTUs) using the *SumaClust* clustering method (Mercier et al. 2013) with a 97% clustering threshold. The most abundant sequence within each cluster was chosen as its representative. Only MOTUs with at least 10 reads in one PCR replicate were retained for taxonomic assignment, which was performed using the *ecotag* program (OBITools v. 2). This program compared each MOTU against a reference database of complete metabarcode sequences obtained from the GenBank public sequence database (version 249). Reference sequences were extracted from GenBank using marker primers and the *ecoPCR* program (Ficetola et al. 2010), allowing for up to three mismatches per primer. Resulting sequences were then dereplicated and filtered to retain only those with taxonomic assignment to at least the family, genus, or species levels.

After taxonomic assignment, an additional data filtering was performed in R using the *metabaR* package (Zinger et al. 2021) to remove MOTUs with a sequence identity < 90% relative to any sequence in the reference database (probable chimeras) and MOTUs whose abundance was highest in at least one negative control replicate (likely contaminants). Additionally, when the relative abundance of a MOTU within a PCR replicate was < 0.1% of its total abundance across the dataset, this relative abundance was set to 0 to mitigate the effect of tag jumps, which occur due to chimeric sequences during the PCR amplification when different tag combinations are combined (Schnell et al. 2015). PCR replicates with fewer than 10,000 reads were removed, and the remaining replicates from the same sample were aggregated. MOTUs with fewer than 100 reads in a sample had their abundance reduced to 0. These steps and thresholds were based on the three replicates containing DNA of known composition (CPOS) to maximize the percentage of sequences corresponding to expected bacterial taxa in the final community (Table S1). The number of reads and MOTUs at each step of data processing and filtration, from raw data to the final results, is shown in Table S2. Despite the filtering methods applied, some MOTUs originating from negative controls could not be fully removed. To avoid artificially inflating the dataset, MOTUs found in the negative controls and those not corresponding to bacterial taxa were discarded. However, we acknowledge that the filtering process was not perfect and decided to use the dataset for analyzing beta diversity, but not alpha diversity, of the bacterial communities.

### 2.5 Statistical Analysis

All analyses were conducted using R 4.2.3 (R Core Team 2022). Fecal microbiota were expressed as relative read abundance, calculated as the ratio of read counts for a given taxon to the total read count per sample.

Microbiota similarity between bird species and/or sites was assessed using Non‐metric Multidimensional Scaling (NMDS) with Bray‐Curtis dissimilarities, applying the square root of relative read abundances to mitigate over‐representation of the most abundant taxa. The analysis was conducted using the *metaMDS* function from the R package VEGAN (Dixon 2003). A stress value below 0.1 was considered indicative of a good spatial representation, while a value above 0.2 indicated poor representation (Clarke 1993). Dispersion was evaluated using distances to the centroid. Centroid coordinates were calculated for each bird species by averaging point coordinates using the *kmeans* function in R. Distances between observation points and their centroids were then computed as the square root of the summed squared differences.

Significant microbiota differences were tested using PERmutational Multivariate ANalysis Of VAriance (PERMANOVA) based on distance matrices, considering seven predictors: bird species, ecology (water or terrestrial bird), body length and trophic guild (see Table 4 for bird characteristics), as well as sampling site, sampling site location (peri‐urban versus rural) and pond surface (see Table 1). These tests were performed using the *adonis2* function from the R package VEGAN. Pairwise comparisons between species or sites were carried out using a permutation MANOVA with the *pairwise.perm.manova* function from the RVAideMemoire R package (Hervé 2020). Differences in dispersion and relative read abundances were analyzed using Levene's test, analysis of variance (ANOVA) with the *aov* function in R, and post hoc comparisons with the *TukeyHSD* function in R.

**TABLE 4:** Characteristics of the nine bird species included in the analysis.

	Mallard	Greylag Goose	Gray heron	Common wood pigeon	Mute Swan	Eurasian coot	Common moorhen	Common Pheasant	Common blackbird
Scientific name	*Anas platyrhynchos*	*Anser anser*	*Ardea cinerea*	*Columba palumbus*	*Cygnus olor*	*Fulica atra*	*Gallinula chloropus*	*Phasianus colchicus*	*Turdus merula*
Ecology	Waterbird	Waterbird	Waterbird	Terrestrial	Waterbird	Waterbird	Waterbird	Terrestrial	Terrestrial
body length (cm)	65	89	98	45	160	39	38	71	27
Main diet (trophic guild)	Omnivorous	Herbivorous	Carnivorous	Herbivorous	Herbivorous	Herbivorous^a^	Omnivorous	Omnivorous	Omnivorous
Migratory status	Potentialy migratory	Primarily migratory	Sedentary	Sedentary and migratory	Sedentary	Primarily sedentary	Sedentary and migratory	Sedentary	Primarily sedentary

^a^ Except during the breeding season, the Eurasian coot is primarily herbivorous. References https://www.oiseaux.net and https://clicnat.fr/ for the regional migratory status.

Potential pathogenic bacteria were identified based on published references on bird pathogens (Grond et al. 2018; Boukerb et al. 2021; Bodawatta, Hird, et al. 2022). Statistical differences in the prevalence of pathogenic genera between fecal samples collected in peri‐urban and rural sites were tested using a Mann–Whitney test, with statistical significance set at *p* < 0.05.

To explore differences in bacterial community composition, cluster heatmaps were constructed using the R package heatmaply (Galili et al. 2018). Host‐bacteria co‐evolution was assessed through phylosymbiosis by correlating host phylogenetic distances (TimeTree; Kumar et al. 2022) with microbiota dendrogram distances via Mantel tests (Mantel 1967). Spearman's rank correlation was applied, with 10,000 permutations, using the *mantel* function from the VEGAN package.

Finally, canonical correspondence analysis (CCA) was used to integrate dietary and microbiota data (Choi et al. 2022). The *cca* function from the R package VEGAN was used with log‐transformed read abundance for overall dataset analysis. Explanatory variables included the seven predictors mentioned above, along with dietary richness in plants and invertebrates, and the invertebrate/plant ratio in the diet. The last two variables were derived from dietary data obtained through metabarcoding, using the same samples as the present study (Fablet et al. 2024). Variable selection for the CCA was refined using bidirectional stepwise regression, and model significance was evaluated with a permutation test of the regression model using the *anova.cca* function.

## 3 Results

The sampling of bird feces was conducted at six localities within the Ramsar site and one on its outskirts (MAI) in Spring 2021 (Figure 1 and Table 1). Bird species identification from fecal samples was performed using metabarcoding in a previous study (Table S3; Fablet et al. 2024). We sequenced part of the hypervariable region 4 (V4) of the bacterial 16S SSU rRNA from 74 fecal samples from 13 bird species (Table S4). However, only 52 samples from nine bird species or families could be included in the dataset for subsequent analyses (Table 2). As a result, the final dataset was limited in size, which constrained the statistical power of our analysis. The removal of 22 samples from the dataset was due to unidentified bird species (*n* = 16), single‐instance species or families (*n* = 5: one common Snipe, *Gallinago gallinago* ; one water rail, *Rallus aquaticus* ; one European Green Woodpecker, *Picus viridis* ; one Rallidae; and one Laridae) and absence of MOTUs in one sample (HAI_008). The 52 identified fecal samples primarily originated from waterbirds (69%), with the Rallidae family (Eurasian coot and common moorhen) accounting for 40% of the total, the Anatidae family (mallard, mute swan and graylag goose) constituting 19%, and gray heron representing 10%. After the first filtration step using the OBITools program suite, the bacterial dataset comprised 13,963 MOTUs. Following the second filtration step, applying the thresholds established from positive and negative controls, 2442 MOTUs remained. Of these, 74% were identified at the family level, 57% at the genus level, and 41% at the species level.

**FIGURE 2:**
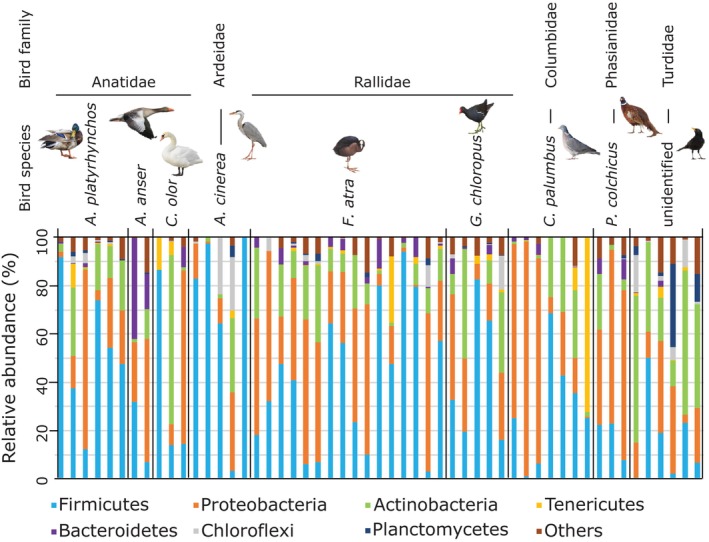
Relative abundance (%) of bacterial phyla in the fecal microbiota of different bird species or families. “Others” represents the sum of phyla with a relative abundance (%) equal to or less than 10% across all bird species.

At the phylum level, the avian fecal microbiota was dominated by Firmicutes and Proteobacteria, with lower relative abundances of Actinobacteria (Figure 2). PERMANOVA analysis indicated significant differences in bacterial composition within the fecal microbiota, with the variation primarily attributed to bird species, explaining 26% of the variance (*p* = 0.005). Among the different bird trait variables tested (ecology, trophic guild, body length, taxonomic order and family), trophic guild accounted for 12% of the variation (*p* = 0.001). However, pairwise comparison tests did not reveal significant differences in bacterial phyla between bird species pairs.

**FIGURE 3:**
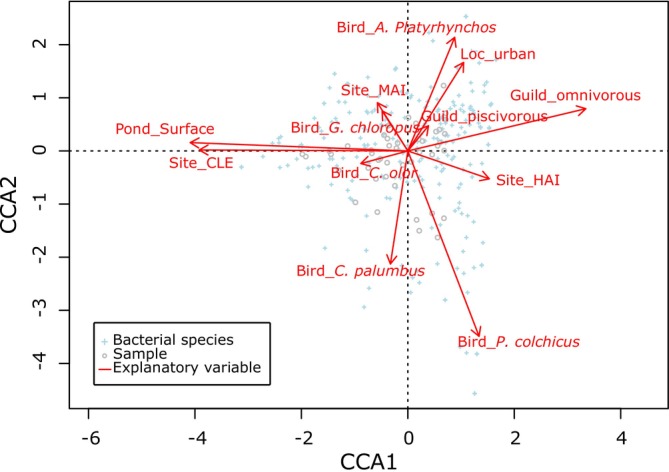
CCA biplot illustrating the relationships between microbiota composition (number of reads per bacterium) and explanatory variables (bird species, trophic guild, body length, site, rural/peri‐urban location, pond surface area, dietary richness in plants and invertebrates, invertebrate/plant ratio). Based on permutation test results for the CCA model axes, the first two canonical axes (CCA1 and CCA2) were statistically significant and contributed to explaining data variance: CCA1, Chi‐squared value = 0.4556, *F*‐value = 2.9543, and *p* = 0.003; CCA2, chi‐squared value = 0.3483, *F* = 2.2587, and *p* = 0.042.

Using CCA, we analyzed the relationship between microbial composition (reads at the genus/species level) and the explanatory dataset, including bird traits (species, ecology, trophic guild, body length) and environmental variables (site, location type, pond surface area, dietary richness in plants and invertebrates and invertebrate/plant ratio in diet) (Tables 1 and 4). The dataset comprised 46 samples with 346 dependent variables and eight explanatory variables. The final model was specified as follows: *log1p(MB) ~ Pond_Surface + Location + Trophique_Guild + Sampling_Site + Bird_Species*, explaining 36% of the variance in the dependent variables. The relationship between dependent and explanatory variables was statistically significant (*R* ^2^ = 0.36, df = 12, *F*‐value = 1.52, *p* = 0.001). These findings suggested that both biological and environmental factors played crucial roles in shaping the microbial communities in bird habitats (see graphical analysis: Figure 3).

**FIGURE 4:**
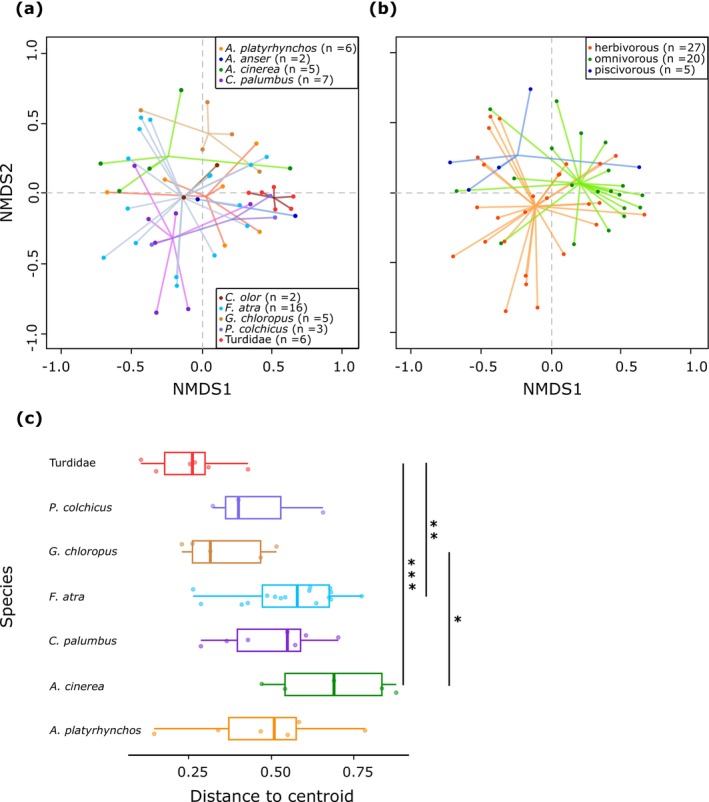
Similarity analysis of fecal microbiota composition at the genus or species taxonomic level using Non‐metric Multidimensional Scaling (NMDS). (a) Comparison among host species. (b) Comparison among host trophic guilds. The quality of the spatial representation, indicated by the stress value, was 0.169. Numbers in brackets indicate the sample size. (c) Dispersion of observations (inter‐individual variation among conspecifics) around their centroid in the reduced NMDS space. Only species with at least three samples are included. Statistical significance is indicated by *p < 0.05, **p < 0.01, ***p < 0.001, based on Tukey's multiple comparisons of means.

We conducted an NMDS analysis to examine fecal microbiota similarities at the genus or species taxonomic level across bird species and traits, with a stress value of 0.169, indicating an acceptable representation (Figure 4). Distinct bacterial community patterns emerged across bird species (Figure 4a) and bird trophic guild (Figure 4b). PERMANOVA tests indicated that differences in bird species and trophic guild explained 24% (*p* = 0.001) and 7% (*p* = 0.001) of the total variance, respectively, with small but significant effects from bird body length and ecology (water or terrestrial birds; Table 5). The pairwise comparison test for significant differences in fecal microbiota between bird species showed dissimilarities mainly between aquatic and terrestrial birds (Table 6). Finally, distances to the centroid indicated intra‐species variation, with Turdidae exhibiting the least dispersed bacterial composition and gray heron the most dispersed (Figure 4c). The ANOVA confirmed significant dispersion differences (*p* = 0.001).

**TABLE 5:** Results of permutation tests examining the influence of host traits and sampling site characteristics on fecal microbiota composition at the genus or species taxonomic level.

Explanatory variable	df	*R* ^2^	*F*	*p*
*Model: ~ Host species × Host Ecology × Host trophic guild × Host body length × Site × Location × Pond surface*				
Host species	8	0.24	1.85	0.001**
Site	6	0.16	1.61	0.001**
*Model: ~ Host Ecology × Host trophic guild × Host body length*				
Host trophic guild	2	0.07	2.05	0.001**
Host body length	1	0.03	1.58	0.028*
Host ecology	1	0.05	2.6	0.001**
Host trophic guild:host ecology	1	0.03	1.57	0.043*
*Model: ~ Location × Pond surface*				
Site localization (urban/rural)	1	0.04	2.57	0.004**
Pond surface	5	0.18	2.04	0.001**

*Note:* A permutation test for Adonis under the reduced model (999 permutations) was performed. Statistical significance is indicated by *p < 0.05, **p < 0.01.

**TABLE 6:** Pairwise comparisons test results for fecal microbiota composition at the genus or species taxonomic level.

Variables	*p*
Host species	
*Anas platyrhynchos* vs. Turdidae	0.018*
*Fulica atra* vs. Turdidae	0.018*
*Gallinula chloropus* vs. Turdidae	0.036*
*Ardea cinerea* vs. *Columba palumbus*	0.030*
*Gallinula chloropus* vs. *Columba palumbus*	0.030*
*Fulica atra* vs. *Phasianus colchicus*	0.018*
*Columba palumbus* vs. Turdidae	0.018*
Sites	
BOU vs. CLE	0.007**
BOU vs. HAI	0.032*
CLE vs. HAI	0.011*
CLE vs. MAI	0.007**
CLE vs. MOR	0.007**
CLE vs. TVA	0.032*

*Note:* A PERMANOVA test (999 permutations) was performed. Only significant pairs are shown. Statistical significance is indicated by *p < 0.05, **p < 0.01.

**FIGURE 5:**
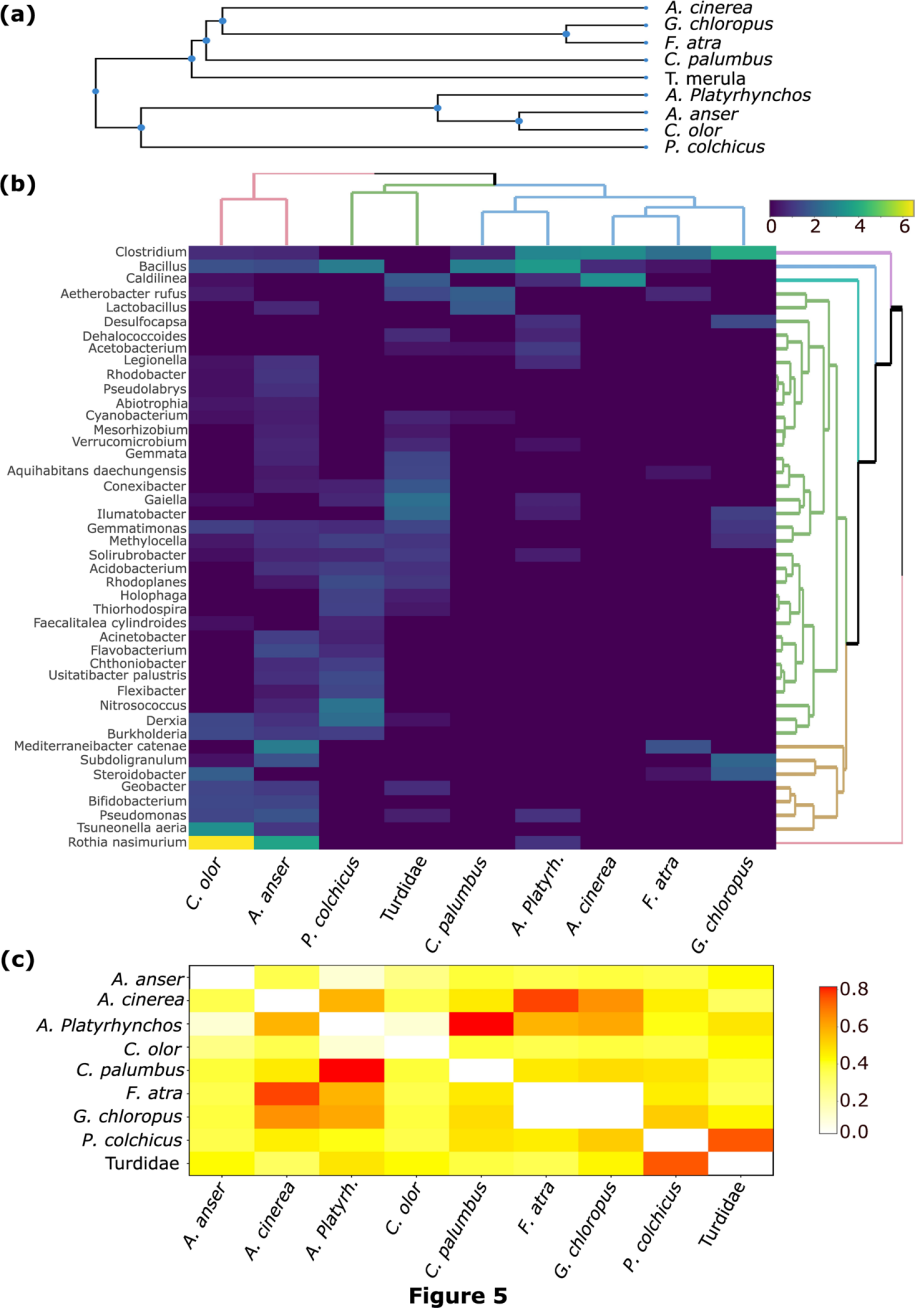
Analysis of the relationship between host phylogeny and fecal microbiota composition: (a) Host phylogeny, (b) Heat map of fecal microbiota composition across bird species, clustered based on the square root of the proportion of reads for the most prevalent bacterial genera. Dendrograms illustrate (i) beta diversity distances among fecal microbiota of different bird species (top), and (ii) bacterial clustering based on similarities in fecal microbiota composition (right). (c) Relative differences between the host phylogeny matrix and the fecal microbiota composition matrix among the different bird species.

Since fecal bacterial composition varied among bird species, we analyzed the relationship between host phylogeny and microbiota composition using a phylogenetic tree of nine bird species (Turdidae was represented by the common blackbird, *Turdus merula* ; Figure 5a). Two major clades emerged: one including Anatids and the common pheasant, and another grouping Rallids, gray heron, common wood pigeon and common blackbird. If fecal bacterial composition is associated with the host phylogeny, species within the same clade should share more bacteria than species in different clades. To illustrate this, we constructed a heatmap depicting the bacterial composition of the nine bird species, focusing on the most abundant bacterial taxa (Figure 5b). At the genus or species level, the microbiota of herbivorous Anatids, specifically the mute swan and the greylag goose, exhibited distinct microbiota characterized by unique clusters: one consisting solely of *Rhotia nasimurium* and another containing *Tsuneonella aeria*, *Pseudomonas*, *Bifidobacterium*, *Geobacter*, *Steroidobacter*, *Subdoligranulum* and *Mediterraneibacter catenae*. In contrast, the microbiota of the other waterbirds was predominantly characterized by the dominance of the genus *Clostridium*, a feature not observed in the terrestrial birds. To test phylosymbiosis, we compared dendrograms illustrating beta diversity distance relationships between bacterial communities among birds (Figure 5b, upper dendrogram) with the phylogeny of the host species (Figure 5a). The Mantel test showed that communities correlate with host phylogeny (Mantel *r* = 0.36; *p* = 0.029), indicating a moderate but statistically significant relationship. Figure 5c highlights the relative differences between the host phylogeny matrix and the fecal microbiota composition matrix among the different bird species. The largest divergences are primarily driven by the positioning of the mallard relative to the other species, the gray heron relative to the rallids, and the grouping of the common pheasant and blackbird together, which may, however, be partly explained by their shared status as terrestrial omnivores.

**FIGURE 6:**
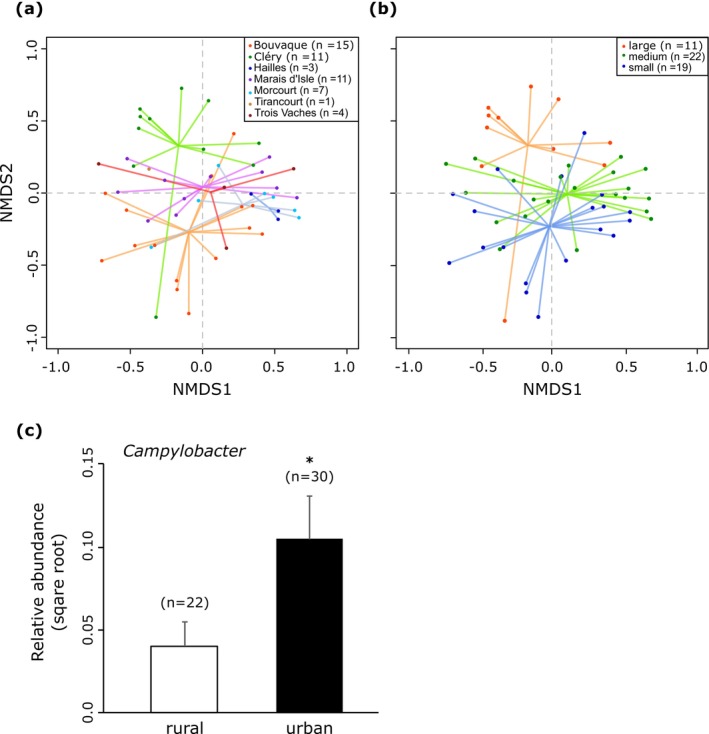
Similarity analysis of fecal microbiota composition at the genus or species taxonomic level using Non‐metric Multidimensional Scaling (NMDS). (a) Comparison among sampling site. (b) Comparison among pond sizes. (c) Prevalence of the potential pathogenic genus *Campylobacter* in bird fecal samples collected from peri‐urban sites. Statistical significance is indicated by *p < 0.05, based on a Mann–Whitney test. Numbers in brackets indicate sample size.

Additionally, NMDS analysis revealed fecal microbiota differences across sampling sites and pond sizes (Figure 6). PERMANOVA tests indicated that sampling sites explained 16% (*p* = 0.001) and pond surface area (ha) explained 18% (*p* = 0.001) of variance differences (Table 5), with peri‐urban versus rural context having a small but significant effect. Notably, among the different potentially pathogenic bacterial genera for birds identified in our dataset (*Campylobacter*, *Clostridium*, *Enterococcus*, *Escherichia*, *Fusobacterium*, *Helicobacter*, *Pseudomonas* and *Streptococcus*), *Campylobacter* was present in 70% of the samples collected in peri‐urban sites compared to 32% in the rural site. This difference in prevalence was statistically significant (Figure 6c; *p* = 0.018). The pairwise comparison test for significant differences in fecal microbiota genera between sampling sites revealed that CLE displayed a notably divergent pattern compared to the other sampling sites (Table 6).

## 4 Discussion

In this pilot study, fecal samples opportunistically collected from seven locations, six within a Ramsar site and one on its outskirts, during Spring 2021 revealed a diverse array of avian fecal microbiota. Metabarcoding analysis identified the bacterial composition from 52 fecal samples from nine bird species or families, predominantly waterbirds, with Firmicutes and Proteobacteria dominating the phylum‐level. Multivariate analyses indicated significant variations in bacterial composition associated with differences in bird species and sampling sites. Notably, significant differences in fecal microbiota were linked to bird body length, ecology, trophic guild, habitat location and pond size. A significant but weak correlation between host phylogeny and microbiota composition was also detected. While the limited sample size restricts the generalizability of specific patterns, the observed ecological trends align with expected ecological and evolutionary patterns. This demonstrates that opportunistic fecal sampling effectively captures key microbial community differences based on bird and habitat characteristics, validating its potential as a reliable tool for exploring broad ecological trends.

### 4.1 Bird Fecal Microbiota Composition

Similar to other vertebrates, the gut microbiota of birds is dominated by four major phyla: Firmicutes, Proteobacteria, Actinobacteria and Bacteroidetes (Waite and Taylor 2014; Grond et al. 2018; Sun et al. 2022). However, birds tend to harbor relatively lower proportions of Bacteroidetes and higher proportions of Proteobacteria (Song et al. 2020). Our results are consistent with these findings but also show that relative abundances of these phyla can vary significantly among bird species and individuals (Figure 2). At lower taxonomic levels, compared to other vertebrates, birds exhibit noticeable differences in gut bacterial composition, clustering separately from humans, insects and fishes while showing greater similarity to bats (Song et al. 2020). At the genus or species level, our data suggested that bird species is a key factor influencing fecal bacterial community structure, despite the absence of a strong association between a specific bird species and a specific microbiota (Figure 4a, Tables 5 and 6). Notably, the mute swan and the greylag goose, as well as the common pheasant and the Turdidae, clustered more distinctly from the other species (Figure 5b). However, this pattern should be interpreted with caution, as the first three species were represented by only two to three samples. Distances to centroids, reflecting inter‐individual dispersions in fecal bacterial composition, also differed significantly between bird species (Figure 4c). Turdidae and the common moorhen exhibited less dispersed fecal bacterial composition, while gray heron and, to a lesser extent, the Eurasian coot displayed the most dispersed patterns. Bird gut microbiota are known to be highly variable among individuals of the same bird species, as they are influenced by both intrinsic factors, such as age, sex, and behavior, and extrinsic factors, including diet, environmental changes, and stochastic processes (Van Dongen et al. 2013; Hird et al. 2014; Trevelline et al. 2020; Bodawatta, Koane, et al. 2021; Góngora et al. 2021; Sun et al. 2022; Wang, Zhong, et al. 2022).

Among the bacterial genera and species identified in the dataset, the most dominant were *Rothia nasimurium* (phylum Actinobacteria), associated with herbivorous anatids; *Clostridium* (phylum Firmicutes), found across all waterbird samples; and *Bacillus* (phylum Firmicutes), present in most bird species analyzed (Figure 5b). The dominance of the genus *Clostridium* in waterbirds has been reported in several studies (Zhao et al. 2017; Laviad‐Shitrit et al. 2019; Wang, Zhong, et al. 2022). This genus encompasses a wide diversity of mostly strictly anaerobic species, some of which have beneficial effects on the host, acting as probiotics and aiding digestion or gut health, while others can be pathogenic. For instance, *C. butyricum* has been shown to promote growth performance, enhance immune function, and support gut microbiota balance in broiler chickens (Yang et al. 2012). Several *Clostridium* species are capable of breaking down cellulose and other complex polysaccharides into metabolites beneficial to the host, such as short‐chain fatty acids (Sabathé et al. 2002; Warnick 2002; Varel and Pond 1992). Among pathogenic species, *C. botulinum* produces the botulinum toxin, which causes botulism, an often fatal neuroparalytic disease in birds, particularly affecting waterbirds and shorebirds (Benskin et al. 2009). Similarly, bacteria from the genus *Bacillus* are common gut symbionts in vertebrates, including wild birds (Xie et al. 2016; Zhao et al. 2017; Wang, Liu, et al. 2018). However, this genus has been extensively studied for its probiotic effects in poultry and other domestic birds, where it has been shown to enhance nutrient absorption, improve metabolic efficiency, modulate immune responses, and reduce pathogen prevalence (Grant et al. 2018; Grond et al. 2018; Bodawatta, Hird, et al. 2022). In contrast, *Rothia nasimurium* has been rarely described in the literature. This species was recently isolated from chickens, where it is considered part of the normal flora but can act as an opportunistic pathogen capable of infecting various animal species, including geese. This bacterium, which exhibits a multidrug‐resistant phenotype, can cause a range of symptoms, including feather loss, and may lead to the death of chickens (Zhang et al. 2022).

### 4.2 Relationship Between Bird Phylogeny and Their Fecal Microbiota

In natural populations across various taxa, including sponges, insects and mammals, correlations have been observed between host phylogeny and interspecific differences in host‐associated internal communities or gut microbiota (Mallott and Amato 2021). This phenomenon, known as phylosymbiosis, relies on heritability through vertical transmission, coevolution, and even adaptation alongside the host. Despite potential confounding factors such as interspecific differences in diet and environment, mixed‐sex animal cohorts or small sampling sizes (Trevelline et al. 2020; Baiz et al. 2023), phylogeny is generally considered a relatively weak predictor of intestinal microbiota diversity in birds overall, possibly due to adaptations related to flight (Song et al. 2020). However, an exception among passerines cannot be ruled out, although results vary across studies (Loo et al. 2019; Bodawatta, Koane, et al. 2021; Baiz et al. 2023).

In waterbirds, a study found that the phylogenic tree of five species, lesser sand plover ( *Charadrius mongolus* ), common redshank (*Tringa tetanus*), brown‐headed gull ( *Chroicocephalus brunnicephalus* ), ruddy shelduck ( *Tadorna ferruginea* ) and bar‐headed goose ( *Anser indicus* ), did not significantly reflect the hierarchical dendrogram of their fecal microbial communities in Tibet wetlands (Bo et al. 2022). Similarly, bird phylogeny did not correlate with gut microbiota in fecal samples from 17 migratory waterbird species in Poyang Lake, China (Wang, Zhong, et al. 2022). Conversely, a significant correlation was found between host phylogeny and intestinal microbiota structure in four resident or migratory waterbird species in Israel, including the black‐crowned night heron ( *Nycticorax nycticorax* ), little egret, great cormorant and black‐headed gull (Laviad‐Shitrit et al. 2019). However, in that study, analysis of intestinal microbiota derived from different sections of the intestine (anterior, middle and posterior) revealed no significant pattern of phylosymbiosis in the posterior intestine region. Given that microorganisms from different parts of the intestine may contribute variably to the fecal microbiota (Grond et al. 2018), these findings suggest that caution is warranted when interpreting phylosymbiosis data derived from fecal samples. Alternatively, other ecological differences among species, such as diet, may lead to parallel patterns between phylogeny and gut microbiota, potentially confounding results, especially when only a few species are considered in study designs. Our data, based on the construction of a phylogenic tree and a dendrogram of the dominant bacteria in fecal microbiota, aligned with the general lack of strong congruence between phylogeny and fecal bacterial community structure in birds (Figure 5). Notably, the mallard duck exhibited a fecal microbiota pattern more divergent from other anatids than from rallids, the common wood pigeon and the gray heron. One possible explanation is the frequent social interactions between mallards and rallids, which may homogenize their fecal microbiota. Similarly, the resemblance between the fecal microbiota patterns of the common pheasant and the common blackbird, despite their phylogenic distance, could partly be attributed to similarities in their omnivorous diets. Host dietary niche has a strong impact on the gut microbiota and has been shown to drive convergence of gut microbiomes across host species with distinct phylogenetic backgrounds throughout the animal kingdom (Mallott and Amato 2021).

### 4.3 Relationship Between Bird Traits and Their Fecal Microbiota

The variability observed in the fecal microbiota within our dataset primarily stems from differences among host species (Figures 3 and 4a, Tables 5, and 6). Host ecology, trophic guild and body length were partially associated with this variability (Figures 3 and 4b, Tables 5, and 6). Previous studies have consistently shown that birds within the same trophic guild tend to harbor more similar gut microbiota, although host species remains the predominant factor (Bodawatta, Koane, et al. 2021; Jarma et al. 2021; Wang, Zhong, et al. 2022). This could partly explain why herbivorous anatids (mute swan and greylag goose) and omnivorous terrestrial birds (common pheasant and Turdidae) exhibit more similar gut microbiota (Figure 5b). A comparative study found that the intestinal microbial networks of carnivorous waterbirds display lower diversity and complexity, reduced integration, and decreased stability compared to those of omnivores (Wang, Zhong, et al. 2022). These characteristics may contribute to greater inter‐individual variability in microbiota composition among carnivores (e.g., gray heron in our study) in response to factors such as diet, age and environment (Figure 4c).

Diet plays a significant role as a primary source of microbial colonizers for the gut, providing nutrients for both the host and the bacterial community while also influencing symbiont selection (Grond et al. 2018; Wang, Zhong, et al. 2022). Beyond trophic guild, which encompasses a broader range of ecological interactions, behaviors, and physiological adaptations than diet, intestinal microbiota may adapt more specifically to the dietary regimes. Tang et al. (2023) demonstrated that seasonal diet shifts in the Sichuan partridge ( *Arborophila rufipectus* ) were accompanied by corresponding changes in gut microbiota composition. Similarly, the Eurasian coot shifts from a predominantly herbivorous diet, consisting mainly of submerged plants, to an omnivorous regime incorporating invertebrates during the breeding season (Brinkhof 1997; Fablet et al. 2024), suggesting that coot gut microbiota may change dynamically across their annual cycle. This dietary flexibility could partly explain the relatively higher individual diversity in fecal microbiota observed in coots compared to other species (Figure 4c). However, in our CCA model, diet, represented by plant and invertebrate richness as well as the invertebrate‐to‐plant ratio, was not identified as a significant explanatory variable.

Migratory birds undergo significant behavioral, physiological and anatomical changes throughout their annual cycle and are exposed to diverse environments, leading to highly variable diets and potentially increased pathogen exposure. This exposure is expected to diversify the sources of their gut microbiota ultimately driving divergence between migrant and resident populations (Wu et al. 2018; Grond et al. 2019; Sun et al. 2022; Wang, Zhong, et al. 2022; Włodarczyk et al. 2024). In Northern France, gray herons are sedentary, Eurasian coots are primarily sedentary, while common wood pigeons exhibit both sedentary and migratory behaviors (Table 4). Thus, the migration status of both Eurasian coots and common wood pigeons may contribute to the inter‐individual variation observed in fecal microbiota in our dataset (Figure 4c).

Our results suggest that body length has a minor but significant influence on the bacterial composition of fecal microbiota (Table 5). One possible explanation is its link to gut length. In several fish families (Cyprinidae and Sparidae), gut length is known to shape gut microbiota structure, with alpha diversity being negatively associated with gut length (Escalas et al. 2021; Liu et al. 2022). A longer gut increases food transit time (see Jackson 1992, on seabirds), which may stabilize the gut environment, reduce microbial turnover, and create more anaerobic conditions that favor anaerobic and facultative anaerobic bacteria, ultimately influencing resource utilization. A similar trend has been observed in passerines, where Bodawatta, Koane, et al. (2021) reported a negative correlation between body mass (used as a proxy for gut retention time) and both microbial diversity and the heterogeneity of cloacal microbiota across 17 species. Birds, due to evolutionary adaptations to flight, have shorter digestive tracts and faster gut retention times compared to non‐flying mammals of similar size (Bodawatta, Hird, et al. 2022). Song et al. (2020) further suggested that bats share gut microbiota similarities with birds due to convergent physiological adaptations to flight. These findings indicate that flight‐related gut adaptations may play a role in shaping gut microbiota structure. Although our dataset did not allow a direct comparison of alpha diversities, differences in gut length may help explain why a larger birds, such as the gray heron, with a longer intestinal tract and potentially longer retention times, exhibit an apparently less diverse gut microbiota compared to Turdidae (Figure 5b).

Because both phylogenetic relationships and ecological traits influence gut microbiota composition, disentangling the factors shaping microbial communities across bird species remains challenging. This complexity is further heightened by the impact of habitat, as suggested by our data and previous studies (Wang, Hong, et al. 2022), which highlight bird ecology as a factor in microbiota composition.

### 4.4 Relationship Between Site and Bird Fecal Microbiota

The gut microbiota of vertebrates is highly responsive to environmental factors, with numerous studies demonstrating its ability to adapt to changes in diet, habitat and human‐induced influences. For instance, the gut microbiota of passerines has been shown to be highly malleable to environmental conditions, including those shaped by human activities such as urbanization (Hird et al. 2014; Teyssier et al. 2018; Knutie et al. 2019; Loo et al. 2019; San Juan et al. 2020). Similarly, significant spatial variation in fecal microbiota composition has been observed in flocks of Lesser black‐backed gulls ( *Larus fuscus* ), where bacterial community richness and diversity were higher in gulls feeding at landfills (Jarma et al. 2024). Expanding on this, altitude has also been shown to influence the intestinal microbiota of species such as brown‐headed gulls ( *Larus brunnicephalus* ) and geese in wetlands, with shifts in bacterial taxa linked to energy metabolism, carbohydrates and amino acids suggesting microbiota adaptations to high‐altitude environments (Wang, Liu, et al. 2018; Bo et al. 2022). Furthermore, the presence of pathogenic bacteria has been associated with differences in diet and habitat in these wetland ecosystems, indicating a possible connection between ecosystem quality and microbiota composition.

Our data revealed that even when birds inhabit the same general area and habitat type, they exhibit significant variability in fecal microbiota composition (Figure 6). This diversity was partially explained by local factors, such as pond size and, to a lesser extent, peri‐urban location (Table 5). The relationship between pond size and bird intestinal microbiota warrants further exploration. However, since pond size in aquatic ecosystems is positively correlated with species richness, particularly of aquatic insects (Oertli and Parris 2019), larger ponds may provide broader trophic niches. Consequently, pond size could indirectly influence gut microbiota diversity by altering dietary inputs. Additionally, structurally complex habitats often support higher biodiversity, which may expose birds to a more diverse microbial environment.

These dynamics were potentially reflected at Cléry‐sur‐Somme (CLE), a large pond in a rural area, that significantly influenced fecal microbiota composition (Figures 3 and 5 and Table 5), offering a broader array of invertebrate and plant resources for birds compared to other sites (Fablet et al. 2024). Notably, this site hosted aquatic insects such as Ephemeroptera from the Caenidae and Baetidae families. These taxa are pollution‐sensitive bioindicators under the AFNOR NF T90‐350 (2004) standard, which is referenced in the European Water Framework Directive. Their presence in bird diets suggests that CLE exhibited higher environmental quality compared to more urbanized sites. Eutrophication and urbanization, which simplify macrophyte and aquatic invertebrate communities, often reduce biodiversity and alter ecosystem dynamics (Rejmankova 2011; Oertli and Parris 2019). However, it is important to note that our CCA model did not identify plant and invertebrate richness or the invertebrate‐to‐plant ratio in the diet as significant explanatory variables, possibly due to our relatively small sample size (*n* = 46).

Finally, the patterns observed in our study may align with broader research on anthropogenic impacts on aquatic systems. For instance, a comparative study in Norway found higher microbial diversity in rural waters less affected by fecal contamination, while urban waters exhibited lower microbial diversity and higher levels of fecal pollution (Paruch et al. 2019). Similarly, our dataset revealed a significantly higher prevalence of the potentially pathogenic enteric bacterium *Campylobacter* in bird droppings sampled from peri‐urban environments compared to rural ones. Given that feeding ecology appears to be a key factor influencing the presence of *campylobacter* in avian intestinal flora (Benskin et al. 2009), this finding supports the idea that urban environments can shape gut microbiota through both direct mechanisms (e.g., exposure to contaminants) and indirect mechanisms (e.g., shifts in diet). Understanding these dynamics is crucial, as waterbirds, which act as reservoirs for *Campylobacter* and other potentially enteric bacterial pathogens, can contribute to the spread of zoonotic diseases, posing significant risks to public health (Benskin et al. 2009; Boukerb et al. 2021).

## 5 Conclusion

Host‐associated gut microbiota profoundly influences the health of birds and aquatic vertebrates, responding rapidly to environmental disturbances. Researchers have suggested that these microbial communities could serve as sensitive indicators for monitoring ecological and physiological changes, advocating for their integration into conservation strategies as essential for preserving biodiversity and enhancing ecosystem resilience (Trevelline et al. 2019; Sehnal et al. 2021). However, bird gut microbiota are influenced by a complex array of traits and environmental factors, leading to intricate patterns whose implications for host health remain largely unknown, particularly concerning potential microbial dysbiosis. It remains unclear whether changes in fecal bacterial communities across hosts and habitat types have functional consequences. Traditional approaches for studying gut microbiota are often invasive, requiring the capture or disturbance of birds, which can induce stress, alter behavior, and disrupt their gut microbiota. Despite being based on a limited number of samples, our findings aligned with expected ecological and evolutionary patterns, suggesting that fecal samples opportunistically collected from the ground may provide a promising, low‐impact alternative. This method could enable extensive assessments of bird gut microbiota, while revealing the relationships between microbial communities, bird traits, habitat and diet.

## 6 Limitations and Future Directions

While our results provide valuable ecological insights, the limited sample size constrains the strength of our conclusions. For some species, the very low number of samples (2–3 individuals) limits the generalizability of observed patterns. Additionally, the explanatory power of our CCA model may have been affected by the relatively small dataset (*n* = 46), potentially masking ecological relationships such as the role of plant and invertebrate richness in diet composition. Future studies should prioritize increasing sample size to enhance statistical power and improve the robustness of findings. Expanding sampling efforts across a broader range of individuals and habitats would allow for more reliable assessments of microbiota variation and dietary patterns.

## Author Contributions

**Laura Fablet:** conceptualization (supporting), investigation (equal), writing – original draft (supporting), writing – review and editing (supporting). **Aurélie Bonin:** data curation (lead), writing – original draft (supporting), writing – review and editing (equal). **Diane Zarzoso‐Lacoste:** conceptualization (equal), methodology (equal), writing – review and editing (equal). **Vincent Dubut:** conceptualization (equal), methodology (equal), writing – review and editing (equal). **Laurence Walch:** conceptualization (lead), formal analysis (lead), funding acquisition (lead), investigation (equal), resources (lead), writing – original draft (lead), writing – review and editing (equal).

## Conflicts of Interest

The authors declare no conflicts of interest.

## Supporting information


**Figure S1.** Per‐base sequence quality plots for the bacterial dataset. The plots show the distribution of Phred quality scores across sample reads, representing the probability of incorrect base calls by the sequencer. A Phred quality score of 20 corresponds to 99% base call accuracy. The graphs display the average, standard deviation and box plot of the quality scores for all reads at each position. PCR reactions were conducted in triplicate.

**Table S1.** Bacterial taxa composition of the mock community (CPOS), their associated MOTUs in the bacterial dataset and a description of the negative controls.
**Table S2.** Number of reads and MOTUs at each step of data processing and filtration using OBITools and the metabaR package, illustrating the progression from raw data to final filtered results.
**Table S3.** Association between each fecal sample, its corresponding bird species and sampling site.
**Table S4.** Taxa read counts retrieved after the last filtering step, along with their taxonomic assignment in each sample.

## Data Availability

The raw sequencing data is publicly available at https://doi.org/10.5281/zenodo.14883869.
