# Supplementary figures and images for: Exploring Bird Gut Microbiota Through Opportunistic Fecal Sampling: Ecological and Evolutionary Perspectives
Source: Ecol Evol. 2025 Apr 14;15(4):e71291. doi: 10.1002/ece3.71291 (PMC11995298; doi:10.1002/ece3.71291)

Replicate 1

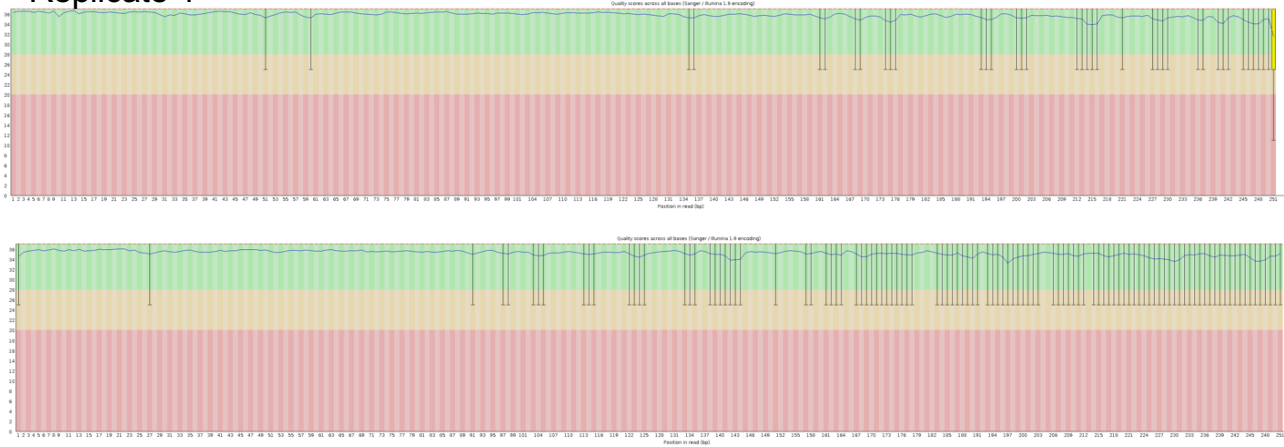

Replicate 2

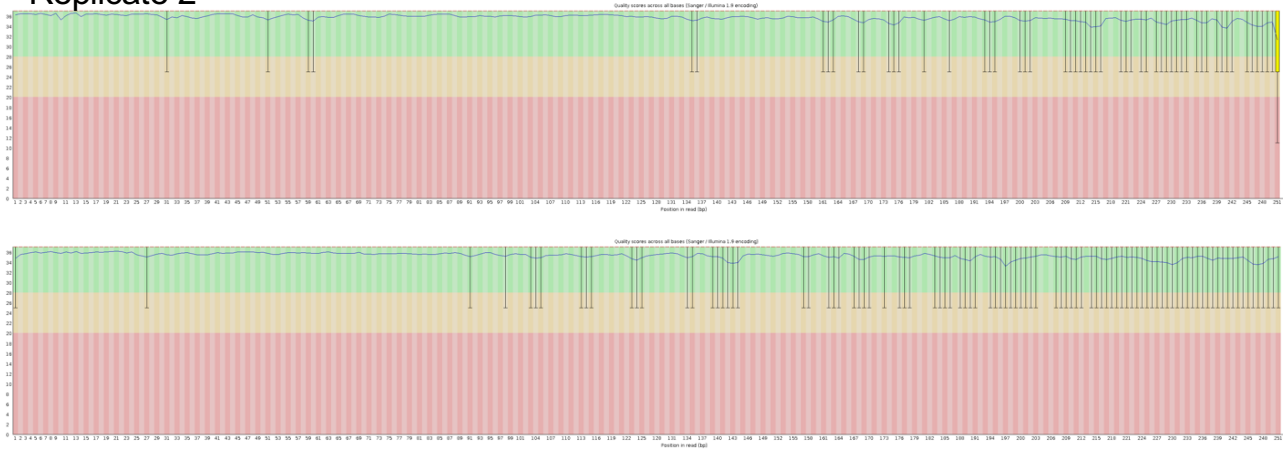

Replicate 3

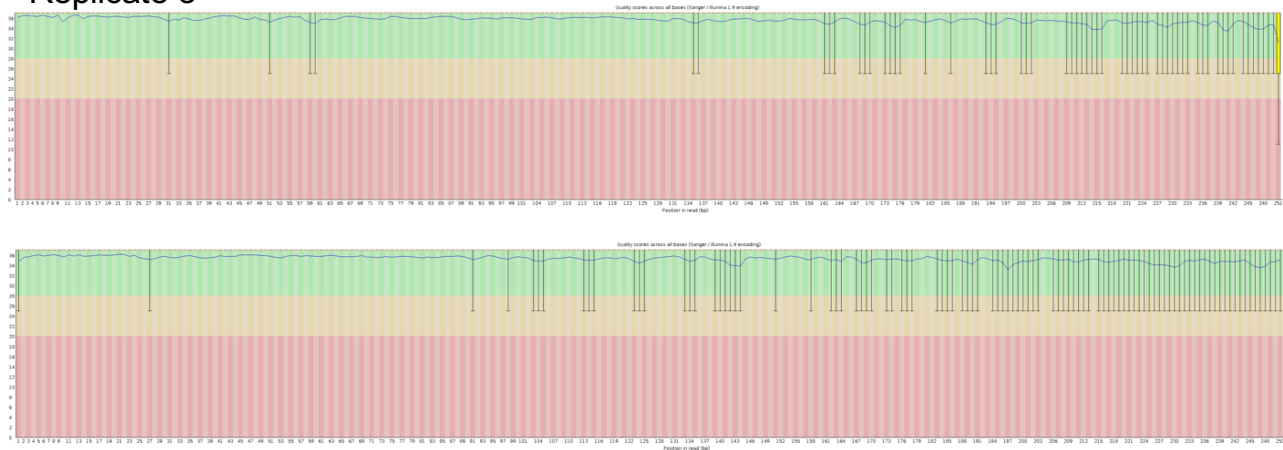

Figure S1

Supplement: Supplementary file 1 — Figure S1. Per‐base sequence quality plots for the bacterial dataset. The plots show the distribution of Phred quality scores across sample reads, representing the probability of incorrect base calls by the sequencer. A Phred quality score of 20 corresponds to 99% base call accuracy. The graphs display the average, standard deviation and box plot of the quality scores for all reads at each position. PCR reactions were conducted in triplicate. [file ECE3-15-e71291-s001.pdf]
